# The Risk for Excessive Anticoagulation With Activated Clotting Time-Guided Monitoring in Patients Undergoing Transcatheter Aortic Valve Replacement

**DOI:** 10.1016/j.shj.2024.100357

**Published:** 2024-09-02

**Authors:** Thijmen W. Hokken, Moniek P.M. de Maat, Nicolas M. Van Mieghem

**Affiliations:** a Department of Cardiology, Erasmus University Medical Center, Rotterdam, The Netherlands; b Department of Hematology, Erasmus University Medical Center, Rotterdam, The Netherlands

**Keywords:** Anticoagulation, ACT, APTT, TAVR

## Abstract

• An activated clotting time <250 ​seconds after an initial unfractionated heparin dose often coincided with sufficient antifactor Xa activity >0.50 U/mL, which may result in additional unfractionated heparin administration, with an increased bleeding risk.
• The point-of-care-activated partial thromboplastin time may be a safer and more reliable anticoagulation monitoring tool during transcatheter aortic valve replacement procedures.

## Current Situation

Transcatheter aortic valve replacement (TAVR) requires large-bore arterial access and is associated with a relevant frequency of intraprocedural thromboembolic neurological events, access site bleeding, and vascular complications.^1^ Adequate anticoagulation is required to prevent clotting and is typically achieved by the administration of intravenous unfractionated heparin (UFH). UFH has a short half-life time and can be neutralized by protamine.^2^ The reference method for monitoring anticoagulation levels on UFH is the determination of antifactor Xa activity (aXa). However, no point-of-care (POC) aXa test is currently available. Historically, the activated clotting time (ACT) has been the preferred method to monitor anticoagulation levels in invasive cardiac procedures because of its ease of use, presumed reliability, and fast processing within minutes. Target ACT levels during a TAVR procedure are empirically set at 250 to 300 seconds.^2^ The poor correlation between ACT and aXa-activity has been reported before in the context of percutaneous coronary interventions and endovascular aortic repair. A study including 3528 patients undergoing percutaneous coronary intervention demonstrated that only 20% of patients reached target ACT levels between 300 and 350 ​seconds, as compared to 86% of patients who reached target aXa-activity level between 0.5 and 1.8 IU/mL.^3^ A retrospective study in 104 endovascular aortic repair patients showed similar results after a UFH bolus of 67 IU/kg. Target ACT value ​> ​250 ​ms was reached in 31%, yet all patients had a target aXa activity >0.50U/mL.^4^

Large-bore access procedures have an inherent risk for (access-site) bleeding. Unnecessary additional UFH administration to achieve target ACT levels in the context of sufficient aXa-activity levels may expose patients to bleeding and (vascular) complications. The poor correlation between ACT and aXa activity shows the unmet need for a reliable POC test that may help reduce procedure-related bleeding events.

## Data Series

In a prospective, observational study, approved by the local institutional ethical committee and conducted in accordance with the Declaration of Helsinki (MEC-2020-0375), we compared POC ACT and activated partial thromboplastin times (APTTs) with aXa activity in 100 consecutive TAVR patients (mean age 78.6 ​± ​7.3 years and 49% male). Target aXa levels for appropriate anticoagulation were set at >0.50 IU/mL.^5^ Target ACT levels during TAVR procedures were >250 ​seconds and during closure <200 ​seconds. Measurements were performed prior to and 30 ​minutes after the first UFH bolus and at the end of the procedure. Sample size calculations are based on the differences in correlation between the ACT POC test and the aXa test and the APTT POC test and the aXa test. The sample size calculation is based on a simulation from the Hotelling’s T-test. The Hotellings T-test measures the (significant) agreement between 2 calculated correlation coefficients and takes the mutual correlation also into account. So, the Hotellings T-test is used to find an agreement between the correlation coefficient of the ACT POC test and the aXa test and the correlation coefficient of the APTT POC test and the aXa test and considers the correlation between the ACT and APTT POC test. Based on the literature, the following Pearson R-correlations are used: a Pearson R-correlation of 0.6 for the ACT POC test and the aXa test, a Pearson R-correlation of 0.85 for the APTT POC test and the aXa test, and a Pearson R-correlation of 0.70 between the ACT and APTT POC test.^6^^,^^7^

We used a 2-sided alpha of 0.05 and a power of 90%. In order to investigate which POC test (ACT or APTT) would provide the best correlation with aXa activity, a sample size of 93 patients is recommended. To allow for a 10% attrition rate in terms of inadequate blood sampling/testing, 100 patients per cohort will be enrolled.

**Figure 1:**
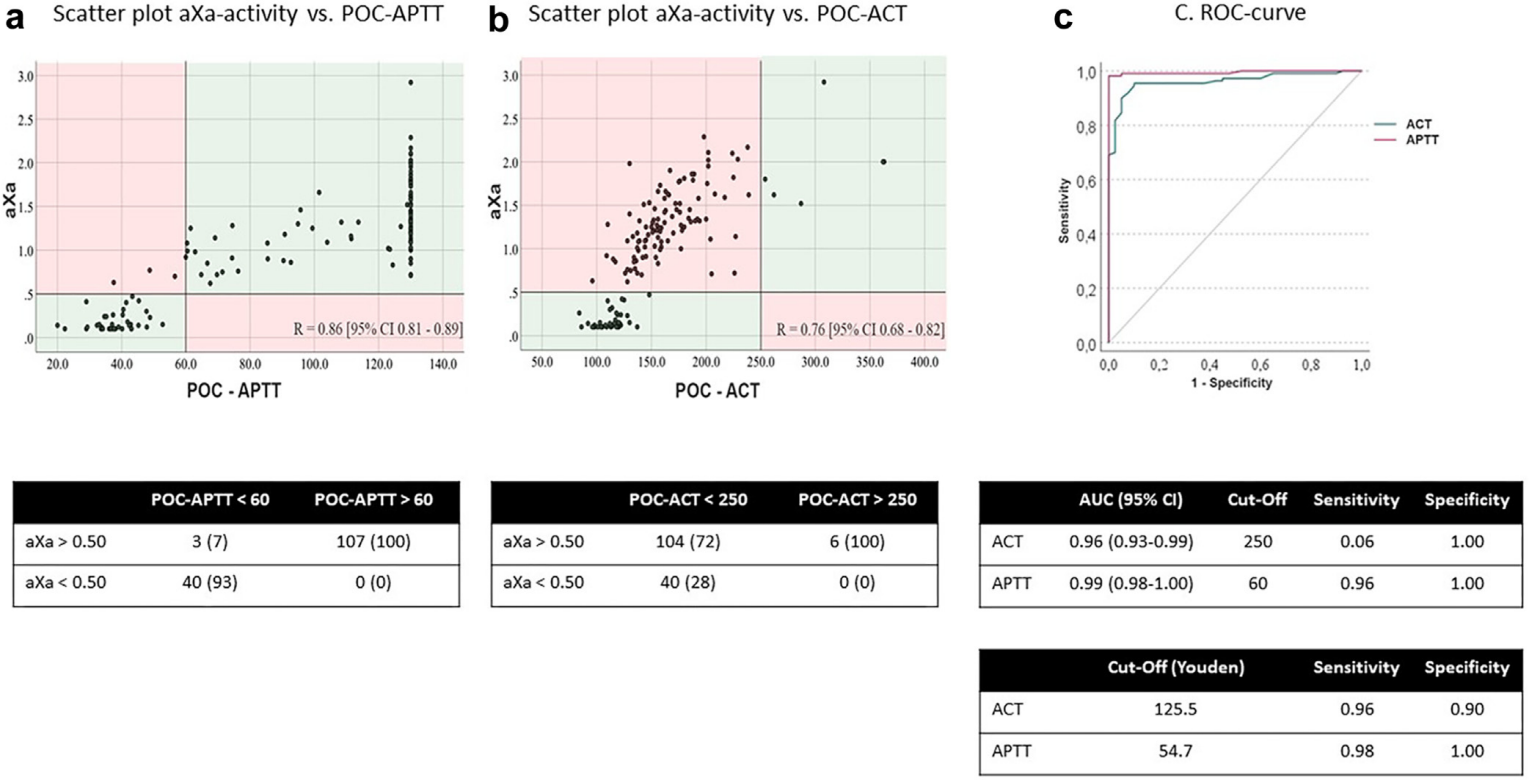
Panel A and B: a scatter plot of (a) aXa-activity vs. the APTT and (b) the aXa-activity vs. the ACT. The green panels visualizes the location of an aXa <0.50 and APTT <60 or ACT <250 and an aXa >0.50 and APTT >60 or ACT >250. The Pearson correlation is included in the figure. Panel C: ROC curve to compare aXa with ACT and APTT with the sensitivity and specificity of the used cut-off points of 60 ​seconds (APTT) and 250 ​seconds (ACT) and the cut-off points based on the Youden criteria Abbreviations: ACT, ​activated clotting time; APTT, ​activated partial tromboplastin time; AUC, area under the curve; aXa, ​anti-Xa activity; POC, ​point-of-care; ROC, receiver operating characteristic.

The Pearson correlation between POC-APTT and aXa was 0.86 (95% CI 0.81-0.89) and between ACT and aXa was 0.76 (95% CI 0.68-0.82). An ACT >250 coincided with an aXa-activity >0.50 U/mL in 100% of the cases. ACT <250 was associated with aXa-activity >0.50 U/mL in 72% of the measurements. A POC-APTT >60 ​seconds coincided with an aXa activity >0.50 U/mL in 100% of the patients. A POC-APTT <60 ​seconds was associated with aXa activity <0.50 U/mL in 93% of the patients and an aXa activity >0.50 U/mL in only 7% (Figure 1a and b). Based on receiver operating characteristic curves to correlate with aXA >0.50 U/mL, a cut-off of 250 ​seconds for ACT (area under the curve 0.96) showed a sensitivity of only 6% (specificity 100%), and a cut-off of 60 ​seconds for APTT (area under the curve 0.99) showed a sensitivity of 96% and specificity of 100% (Figure 1c). Based on the Youden criteria, the best cut-offs were 125.5 for ACT (sensitivity 96%, specificity 90%) and 54.7 for APTT (sensitivity 98%, specificity 100%).

We applied linear mixed effect (LME) models to correlate ACT and APTT results (two separate, non-nested models) as independent variables with aXa concentration as dependent variable. The LME methodology accounts for clustering of data within patients. The Akaike information criterion was determined to compare test results. A lower Akaike information criterion represents the best-fitted model (based on a maximum log likelihood). We also performed the Vuong’s test to calculate the *p* value for the best fit with aXA activity.

The LME model was 158.9 between aXa activity and ACT and 78 between aXa activity and APTT. The Vuong test confirmed the superior correlation between aXa activity and APTT (*p* = 0.01).

## Conclusion

Our findings demonstrated superior correlation of aXa activity with POC-APTT than with ACT. More specifically, an ACT <250 ​seconds after an initial UFH dose often coincided with sufficient aXa activity >0.50 U/mL and additional UFH administration to reach ACT >250 seconds could result in excessive anticoagulation and incremental bleeding risk. The POC-APTT may be a safer and more reliable anticoagulation monitoring tool during TAVR procedures. Potential (unmeasured) confounders to take into account were the timing of administration of the first heparin bolus, exact patient weight, and prior antithrombotic therapies. The decision to react to the measured ACT levels was per the operator’s discretion. This proposition should be validated in a properly sized randomized controlled clinical trial that evaluates bleeding and thromboembolic events with anticoagulation monitoring with ACT vs. POC APTT during TAVR procedures.

## Ethics Statement

This study is conducted in accordance with the declaration of Helsinki. The study was approved by the local ethical committee (MEC-2020-0375).

## Funding

Roche Diagnostics supplied the CoaguChek Pro II and the cuvettes free of charge. There was no specific financial funding involved in this trial.

## Disclosure Statement

N. Van Mieghem has received research grants from Abbott Vascular, Biotronik, Boston Scientific, Medtronic, Edwards Lifesciences, Abiomed, PulseCath BV, Pie Medical, and Daiichi Sankyo. The other authors had no conflicts to declare.
